# Effects of regional location on the genotype and phenotype of historical Irish brewing yeast

**DOI:** 10.3389/fmicb.2025.1452334

**Published:** 2025-03-11

**Authors:** Daniel W. M. Kerruish, Paul Cormican, Elaine M. Kenny, Carl J. M. Whelan, Steve Gilsenan, Eibhlin Colgan, Katherine A. Smart, Chris A. Boulton, Sandra N. E. Stelma

**Affiliations:** ^1^TU Dublin, School of Food Science and Environmental Health, Grangegorman, Dublin, Ireland; ^2^Diageo Ireland, St James’s Gate, The Liberties, Dublin, Ireland; ^3^ELDA biotech, Kildare, Ireland; ^4^Brewing Consultant, Burton upon Trent, United Kingdom

**Keywords:** *Saccharomyces cerevisiae*, historical Irish brewing yeast, brewing, phylogenetic, domesticated microbes

## Abstract

Most commercial beers are made using water, malted barley, and hops as the principal ingredients and *Saccharomyces* yeast as the transforming microorganism. The yeast is used in a semi-conservative process in which crops are collected from one fermentation, stored, and a proportion recycled into a subsequent fermentation. This process differs from wine, cider, and spirit manufacturing where the yeast culture is only used once. The serial fermentation process is continued approximately 8–12 times after which a new culture of verified purity and identity is introduced. This increases the likelihood that the yeast remains true to type. Many commercial brewers use proprietary strains the origins of which are usually unknown. Advances in genetic analyses provide a means for probing the origins of brewing yeast strains, and in this study, six historical Irish brewing yeasts from five breweries located within Ireland were assessed. Using Illumina sequencing technology, whole-genome sequencing data were generated. Single nucleotide polymorphism analysis of these data established that the historical Irish brewing yeast group falls within the previously described “Britain” subpopulation Beer 1 clade. Further analysis established that the six historical Irish brewing yeasts separate into two subgroupings, which associated with specific regional locations. Furthermore, the assessment of the six historical Irish brewing yeast phenotypic attributes relevant to brewing correlated within the same regional location groupings. Our data provide further evidence of how brewing requirements associated with specific beer styles have influenced yeast strain selection.

## Introduction

Beer is made principally from hops, water, and malted barley (Stewart et al., 2017). Yeast, usually strains of *Saccharomyces*, is the catalyst which as a result of its growth and metabolism converts the starting material, wort, into beer. For reasons of consistency and finance, yeast is cropped at the end of one fermentation and a proportion is retained and used to initiate the next fermentation (Lawrence et al., 2012; Powell et al., 2004). Care is taken to ensure that the yeast remains contamination-free and fit for purpose (Boulton and Quain, 2008). To ensure that the yeast remains true to type, the yeast culture is replaced typically after 8–12 brews or more frequently when using highly concentrated worts, where the risk of formation and selection of mutants is elevated (Kalayu, 2019). Replacement yeast at a concentration suitable for commercial brewing is produced through a process termed propagation and is derived from a master culture of verified identity and purity (Boulton and Quain, 2008; Moutsoglou and Dearden, 2020; Smart, 2007). Propagating yeast from a stored master culture minimizes the risk of genetic drift, and the phenotype of the yeast remains consistent (Briggs et al., 2004). For many long-established commercial brewers, proprietary yeast strains are used, which often were originally selected historically from wild populations or obtained via other routes, such as sharing between different brewing companies. Commonly, the actual origins remain unknown.

These wild populations of yeast have, through human selection, become domesticated in a process similar to that which occurred with plants and animals (Steensels et al., 2019). The process of domestication is not limited to microbes associated with beer fermentation. Microbes required for the manufacture of yoghurt, soy sauce, sake, and cheese have also been selected to conserve advantageous phenotypes (Steensels et al., 2019). For example, the fungus *Aspergillus oryzae* used to make soya bean paste, sake, and soy sauce shares a 99.5% similarity in coding regions to the aflatoxin-producing fungus *A. flavus*. However, the domesticated *A. oryzae* remains non-aflatoxigenic despite containing the aflatoxin biosynthetic gene cluster (Kusumoto et al., 2000). Consequently, the phenotype of *A. oryzae* has arisen, at least in part, as the result of the effects of domestication (Machida et al., 2008). Similar conclusions can be made with respect to the transforming microbes associated with wine and cider fermentation, cheese production, and yoghurt manufacturing, as all exhibit phenotypic functionality related to domestication (Campbell-Sills et al., 2015; Hao et al., 2011; Passerini et al., 2010).

Ales and stout beer styles are brewed using strains of the yeast *Saccharomyces cerevisiae* (Gonçalves et al., 2016). Strains of *S. cerevisiae* yeast have been used by humans for millennia to produce alcoholic beverages—wine, cider, distilled spirits, and sake—as well as to leaven bread and latterly for bioethanol production (Gallone et al., 2016; Peter et al., 2018). Phylogenetic studies of *S. cerevisiae* used in manufacturing generally place individual strains within their respective industrial applications (Gallone et al., 2016), with beer yeast grouping together; similarly, yeasts used to make wine, sake, and bioethanol form other distinct groups (Gallone et al., 2016; Gonçalves et al., 2016; Peter et al., 2018). Many of these industrial brewing *S. cerevisiae* yeasts have domesticated phenotypic qualities, such as reduced environmental fitness, reduced sporulation activity, and increased utilization of the sugar maltotriose, a sugar found in unfermented beer (wort) (Gallone et al., 2016; Peter et al., 2018).

Ireland has a rich and unique brewing history, producing and originating two notable styles: Irish ale and Irish dry stout (Cornell, 2003; Foster, 2014). Strains of *S. cerevisiae* were used to brew these beers (Kerruish et al., 2024). In this study, a total of six historical Irish brewing yeasts were assessed from five historical Irish breweries. These breweries were Smithwick’s (Kilkenny brewery 1710–2014), Macardle (Macardle Moore brewery 1863–2001), Cherry (New Ross brewery 1828–1956), Perry (1800–1966), and the Great Northern Brewery (1896–1956). Following the closure of the Smithwick’s and Macardle Moore breweries, production of these beers were transferred to the Guinness brewery, St James’s Gate, Dublin. Macardle Moore and Great Northern breweries were both located within the city of Dundalk, whereas the Smithwick’s, Cherry, and Perry breweries were situated further south in the contiguous counties of Laois, Kilkenny, and Wexford.

To understand the relationship between the six historical Irish brewing yeasts and their association with other industrial *S. cerevisiae* strains, molecular and phenotypic assessments were conducted. The aim was to investigate the origins of the historical Irish brewing yeasts based on the presence of specific single nucleotide polymorphisms (SNPs) and whether, or not, these could be correlated with specific regional locations. These historical Irish brewing yeasts grouped within the previously described Beer 1 clade, with ancestral lineage annotated to the “Britain” subpopulation. Furthermore, the phenotypic assessment, using standard brewing phenotypic quality assurance methods, of the historical Irish brewing yeasts established that there are significant differences in brewing-related phenotypic characters between the different yeasts, but that these differences are decreased when the yeasts are grouped by their respective regional origins. Consequently, yeasts used to brew different beer styles are phenotypically similar, whereas yeasts used to brew specific beer styles are phenotypically more diverse. The data presented in this study highlight the suggestion that brewing requirements influenced yeast selection, emphasizing the role of human domestication on microbes.

## Methods

### Yeast strain selection and maintenance

A total of six Irish historical brewing yeasts were selected for assessment (Table 1) from five Irish breweries. The yeasts included the current production of Smithwick’s yeast and five yeasts from four other historical Irish breweries that are no longer used to produce beer commercially. Cultures were stored in cryo vials (Fisher) in liquid nitrogen at −196°C using 50% glycerol (Sigma-Aldrich) as a cryopreservative. Before liquid nitrogen storage, all historical yeasts were deposited into the library on wort agar slopes and stored in a refrigerator at 4°C. To ensure the yeasts remained viable and free of contamination, the yeasts were recultured every 6 months (Kerruish et al., 2024). Cultures were recovered and inoculated into 25-ml tubes containing 10 mL of YPD (10 g L^−1^ yeast extract, 20 g L^−1^ peptone, 20 g L^−1^ glucose) (Oxoid) and incubated at 25°C in an orbital shaker (Stuart Scientific) at 120 rpm for 24 h. Serial dilutions of 100 μL of cultures were spread-plated onto Wallerstein Nutrient Agar (Oxoid) and incubated at 25°C for 12 days following the European Brewery Convention Yeast Giant Colony Method 3.3.1.1 (Richards, 1967). At the end of the incubation, three single yeast colonies were selected for DNA extraction based on morphological similarity.

**Table 1:** Name and description of yeast strains used in this study and selected following a literature review of the Guinness archives.

Yeast	Naming reference	Source	Brewing group	Location co-ordinates
Cherry	Cherry 1960	Cherry’s pitching yeast	Cherry	Lat: 52.3936 Long: −6.9477
Macardle 1965	Macardle 1965	1965 Macardle pitching yeast	Macardle Moore	Lat: 53.9807 Long: −6.3801
Macardle 1993	Macardle 1993	1970 Smithwick’s pitching latterly used as Macardle pitching yeast	Macardle Moore	Lat: 53.9807 Long: −6.3801
Great Northern Brewery 1958	GNB 1958	Great Northern Brewery stout pitching yeast	GNB	Lat: 54.0001 Long: −6.4145
Perry	Perry 1967	1967 Perry pitching yeast	Perry	Lat: 52.8560 Long: −7.584
Smithwick’s*	Smithwick’s 1986	1986 Smithwick’s production yeast	Smithwick’s	Lat: 52.654 Long: −7.2539

*Denotes yeast presently used in commercial beer production.

### DNA extraction and interdelta yeast typing

Three giant colonies of each culture were selected and transferred to microfuge tubes containing 700 μL of molecular-grade water (Fisher). Yeast cells were recovered by centrifugation, and DNA was extracted following the manufacturer’s guidelines using a PureLink Microbiome DNA Purification Kit (Invitrogen). Individual strains were identified using the interdelta polymerase chain reaction (PCR) method (ASBC, 2008; Legras and Karst, 2003), with primers δ2 (5′-GTGGATTTTTATTCCAAC-3′) and δ12 (5′-TCAACAATGGAATCCCAAC-3′), using a BioRad T100 Thermocycler and Invitrogen’s Platinum Hot Start PCR Master Mix. PCR products were analyzed on an Agilent 2100 Bioanalyzer using the Agilent DNA 7500 chip. The resulting bands were analyzed using Minitab 19 Statistical Software (2019) hierarchical clustering function, with dendrograms produced using the Euclidean distance function.

### Illumina whole-genome sequencing and *de novo* assembly

The interdelta sequence results were used to select yeasts for whole-genome sequencing with typical Inter delta sequence banding used as the selection criterion for the Irish brewing yeasts. In total, six historical Irish brewing yeasts were subjected to whole-genome sequencing performed by ELDA biotech (Kildare, Ireland). Yeast samples were subcultured onto Wallerstein Nutrient Agar (Oxoid), and single colonies were picked for DNA extraction using the Thermo Scientific Yeast DNA Extraction Kit (Thermo Scientific). Extracted DNA was analyzed using a Qubit (Thermo Fisher Scientific) to determine dsDNA content. Aliquots of 1 ng of DNA were used as input for library preparation using the Illumina Nextera XT DNA library prep protocol with no deviations. Stock libraries of 1–4 nM were generated, and samples were pooled for sequencing and denatured according to the manufacturer’s instructions for loading on the Illumina MiSeqor NovaSeq sequencer. Samples were sequenced to a minimum depth of 30X coverage using 2 × 250 bp paired reads. All samples were quality checked for low-quality sequence bases and the presence of adapter contamination using Trimgalore (Version 0.6.1). All identified adapters were cleaved from both the forward and reverse sequencing reads, and those with runs of low-quality bases were trimmed using a Phred scale cutoff of 10. All samples were aligned to the reference genome *S. cerevisiae* S288c (http://downloads.yeastgenome.org/sequence/S288C_reference/genome_releases/S288C_reference_genome_R64-1-1_20110203.tgz) using Burrows-Wheeler Alignment (Version 0.7.17; Li and Durbin, 2009). Alignments were sorted and duplicate reads were identified and marked for exclusion from downstream analysis using Samtools (Version 1.10) (Danecek et al., 2021). Alignment metrics for each sample were collated using Qualimap (Version 2.2.1) (Okonechnikov et al., 2016). All samples were assembled *de novo* using Spades (Version 3.14). For each sample, all contigs shorter than 500 base pairs in length were discarded. A reference-guided scaffold of each assembled sample genome against the *Saccharomyces cerevisiae* S288C genome sequence was generated using Ragtag (v. 1.0.2) (Alonge et al., 2019). Artificial padding of “N” characters was placed between the reference scaffolded contigs. All bioinformatic software used in this study is specified in Supplementary Presentation 1.

### Determination of the historical Irish brewing yeast phylogeny

Sequencing data for 176 *S. cerevisiae* samples, including 154 retrieved *S. cerevisiae* samples from NCBI (BioProject PRJNA323691), were assessed. All retrieved samples were quality checked for low-quality sequence bases and the presence of adapter contamination using Trimgalore (Version 0.6.1). All identified adapters were cleaved from both the forward and reverse sequencing reads, and reads with runs of low-quality bases were trimmed using a Phred scale cutoff of 10. All samples were aligned to *S. cerevisiae* S288c reference genome using BWA mem (version 0.7.17) (Li and Durbin, 2009). Alignments were sorted, and duplicate reads were identified and marked for exclusion from downstream analysis using Picard (Version 2.18.23). Alignment metrics for each sample were collated using Qualimap (Version 2.2.1) (Okonechnikov et al., 2016). The misalignment of reads in original BWA alignments was corrected using Genome Analysis Toolkit (Version 4.1.4-1) (Waterhouse et al., 2018) with the base score recalibration performed on the corrected alignments. Single Nucleotide Polymorphism and Indel discovery and genotyping were performed across all 176 samples simultaneously with GATK used to filter sites based on the following metrics: quality score >30, mapping scores >40, and read position rank sum <8. All individual genotypes with less than 10X coverage were set to uncalled. Annotation and effect prediction for each variant was estimated using SnpEff (Version 4.3) (Cingolani et al., 2012).

Orthologous genes across all assembled genomes were inferred using Orthofinder (Version 2.3.3) (Emms and Kelly, 2019). Sequences from orthologous genes were concatenated and aligned using MULtiple Sequence Comparison by Log-Expectation (Version 3.8.31). A phylogenetic analysis of the concatenated alignment of data from all orthologous genes was performed using the maximum-likelihood approach implemented in RAxML (Version 8.2.4) (Purcell et al., 2007) based on the GTRGAMMA model of sequence evolution and a rapid bootstrap analysis for 1,000 bootstrap replicates. The tree which was rooted using the outgroup species *S. paradoxus* was visualized and annotated using the ggtree (Yu et al., 2018) package in R.

FastSTRUCTURE (Version 1.0) (Raj et al., 2014) was used to quantify the number of populations and the degree of admixture in the genomes examined in this study. Owing to the high degree of sequence similarity between the Guinness samples, a single representative sample (IDS1) was used in this analysis; consequently, admixture in 161 genomes was assessed. The full set of biallelic segregating sites identified across all samples was filtered based on a minor allele frequency (MAF) <0.05 and SNPs in linkage disequilibrium, using PLINK (v1.09) (Boeva et al., 2012). FastSTRUCTURE (Raj et al., 2014) was run on a filtered set of SNPs, varying the number of ancestral populations (K) between 1 and 10 using the simple prior implemented in fastSTRUCTURE (Raj et al., 2014), with *K* = 8 found to be optimal.

### Copy number variation

Analysis of the heterozygous bi-allelic SNPs for each historical Irish brewing yeast established variable copy number across the chromosomes, and subsequently CNV was normalized against an appropriate background copy number for each yeast. In addition, CNV was estimated in 250 base pair non-overlapping windows across the entire ~12 million bases of the *S. cerevisiae* genome using Control-FREEC (Version 5.7) (Boeva et al., 2012). Plots depicting CNVs for the historical Irish brewing yeasts were generated in R using publicly available code (Fijarczyk et al., 2020).

### Sporulation

The sporulation potential of the different historical Irish brewing yeasts was assessed using the ASBC Yeast 7 Sporulation Method (ASBC, 1985). A total of 1,000 cells per sample were examined using a Nikon Eclipse C*i* microscope with 100× magnification. Ascospores stained green to blue-green, while vegetative yeast cells stained pink to red. Independent triplicate analyses were performed for each yeast. The incidence of sporulation was expressed as a percentage.

### Assessment of fermentation properties

Fermentation ability was assessed using 180-ml mini-fermenters (Fisher) containing 120 mL of 12^o^P wort. Cultures were recovered from liquid nitrogen and sufficient yeasts for the experiments generated by successive serial aerobic incubations in 10-ml Yeast extract Peptone Dextrose, 90-ml 12^o^P wort, and 900-ml 12^o^P wort. A single batch of all-malt-hopped wort was used for all experiments to eliminate batch-to-batch variation. Wort was produced in the Guinness pilot plant and stored at −20°C in 5-L aliquots. Before use, it was thawed and sterilized by autoclaving.

Yeast cells were recovered by centrifugation and washed three times by successive suspension in distilled water and recentrifugation. The viability and yeast cell concentration of each culture was determined using the EBC methods, EBC 3.1.1.1 Hemocytometer (Smart et al., 1999) and EBC 3.2.1.1 Methylene Blue (Buckee and Mundy, 1994). Triplicate fermentations were inoculated with 1 × 10^7^ viable yeast cells per ml into 180-ml mini-fermenters containing 120 mL of air-saturated 12^o^P wort. Fermentations were incubated at 25°C and stirred continuously using a stirrer plate (mix 15 eco plate Camlab) set at 250 rpm. Mini-fermenters were sealed with a butyl rubber plug secured with an aluminum cap (Fisher) and fitted with a Bunsen valve to allow CO_2_ to be released. Fermentation progression was measured by periodically monitoring weight loss. The endpoint was established when three successive identical readings were recorded.

### Analysis of fermentation metabolites

Concentrations of selected yeast-derived flavor compounds were measured using a gas chromatographic procedure using a modified version of the EBC Vicinal Diketone method Analytica-EBC Method 9.24.2 (Buckee and Mundy, 1994). End-fermentation samples (30 mL) previously clarified by centrifugation were transferred to McCartney bottles, esters and higher alcohol concentrations determined using a Flame Ionization Detection detector. Peak areas for the metabolites were normalized using appropriate internal standards.

### Analysis for phenolic off-flavor (4-vinyl-guaiacol, 4-VG)

The ability of yeasts to produce 4-vinyl-guaiacol was determined according to Analytica-EBC Method 2.3.9.5 (Analytica-EBC, 2018) phenolic off-flavor (POF) method using gas chromatography–mass spectrometry. Washed yeast samples were inoculated at a concentration of 1 × 10^6^ viable cells.ml^−1^ into 25-ml tubes containing 10 mL of YPD medium supplemented with 0.1 mL of ferulic acid (hydroxycinnamic acid) solution. Triplicate incubations were performed for each yeast. After incubation at 25°C for 48 h, 5 mL was transferred to an autosampler vial (Fisher) containing 2 μL of the internal standard of 4-vinyl-guaiacol (Sigma-Aldrich). Analyses were performed using an Agilent 6890/7890 GC system fitted with a Zebron ZB-Wax 60.0 m × 250.00 μm × 0.25 μm column. The initial oven temperature was 60°C. After 10 min, this was increased to 220°C at a rate of 10°C min^−1^ and then held for 2 min. Moreover, 4-vinyl-guaiacol concentration was determined using an Electron Capture Detector detector with a temperature of 150°C and a makeup flow rate of 60 mL min^−1^ (helium gas). The peak area for 4-vinyl-guaiacol was normalized against the internal standard.

### Alcohol concentration

Ethanol concentration was determined using near-infrared spectroscopy with an Anton Paar Alcolyser (anton-paar.com), following the manufacturer’s guidelines.

### Sugar concentration

Samples were analyzed using an Agilent 1260 Infinity II system with a refractive index detector (Infinity II 1260 WR RID) and a Zorbax Carbohydrate Column (4.6 × 250 mm, 5 μm, P/N: 840300-908). The other acquisition conditions were as follows: mobile phase was a 70/30 mix of acetonitrile and water; sample injection volume was 50 μL; flow rate was isocratic and set at 1.5 mL min^−1^. The column oven was kept at a constant 35°C. Samples were bracketed on either side with freshly made known standards.

### Flocculation

Flocculation was assessed using the EBC Gilliland Method EBC 3.5.31 (Gilliland, 1951). The EBC method uses visual inspection of flocculation behavior, categorizing the yeasts using prescribed classifications: Class 1, non-flocculent; Class 2, slightly flocculent; Class 3, moderately flocculent; Class 4, highly flocculent. An addendum to the EBC method was the addition of four control yeasts representing the different classifications.

### Statistics and reproducibility

Statistical analyses were performed using Minitab 19 Statistical Software (2019) and Xlstat 20 Excel Statistical Package (2020). Mini-fermentations were performed using three independent biological replicates and the statistical significance of ethanol production, fermentation metabolites, and POF was determined using one-way ANOVAs.

## Results

### Determining the origins of the historical Irish yeast

**Figure 1:**
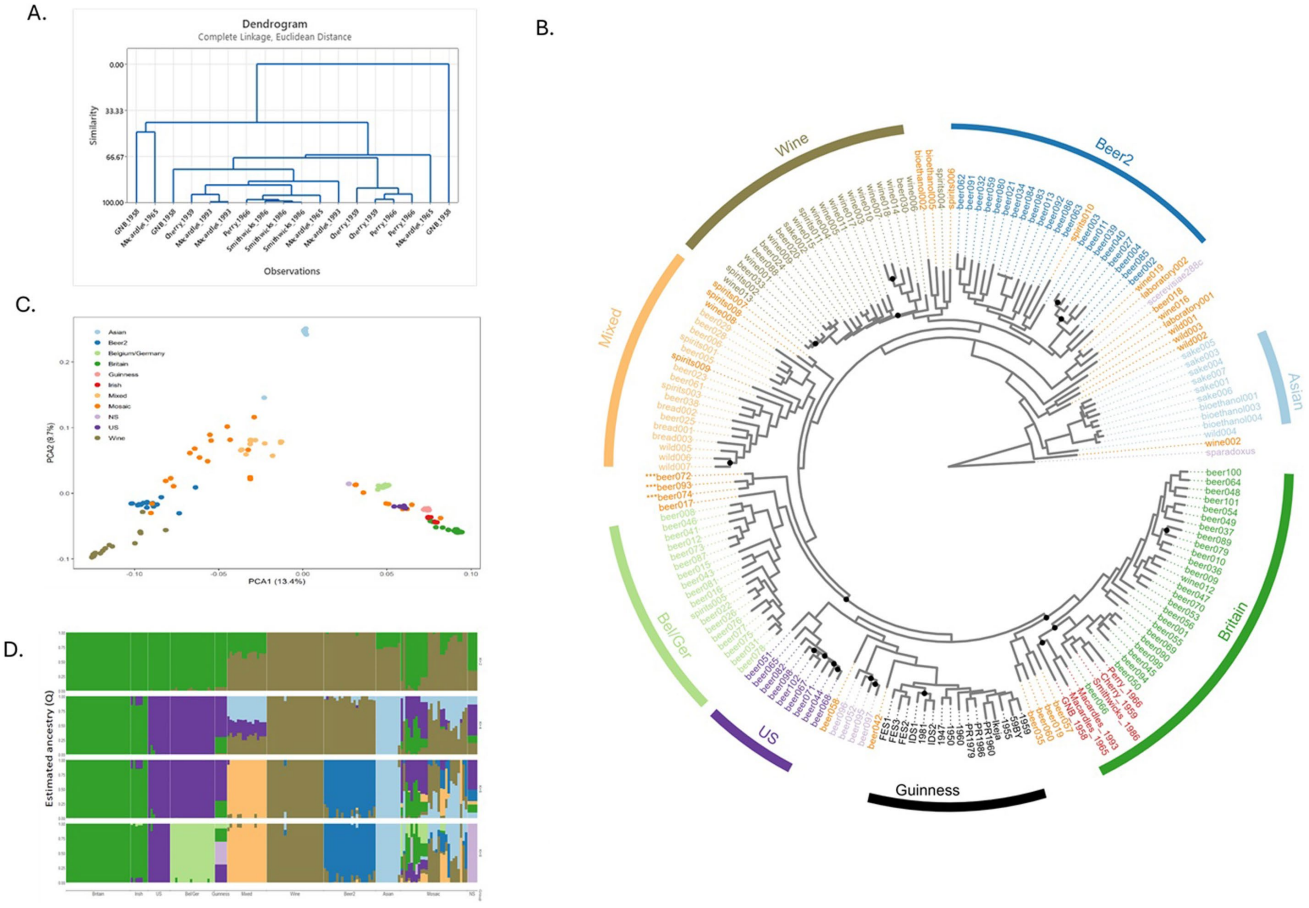
Phylogeny and population structure of the historical Irish brewing yeasts and other industrial *S. cerevisiae* strains. **(A)** Hierarchical clustering analysis of PCR products, determined using Bioanalyzer 2.0 DNA chips, of the interdelta specific primers δ2/δ12 (Legras and Karst, 2003). PCRs were conducted on typical and atypical historical Irish brewing yeast morphology as observed through Giant Colony Morphology assessment. **(B)** Historical Irish brewing yeast within the maximum-likelihood phylogenetic tree of *S. cerevisiae*. Historical Irish brewing yeasts were sequenced using an Illumina MiSeq platform and combined with 170 previously sequenced *S. cerevisiae* (Kerruish et al., 2024). Branch length reflects the number of substitutions per site, with color denoting the yeast lineage. A maximum-likelihood (ML) phylogenetic tree was reconstructed in RAxML v8.2.4 (Stamatakis, 2014), performing 100 iterations to search for the best tree, using a discrete GTRGAMMA model of rate heterogeneity. Bootstrap branch support was assessed by performing 1,000 pseudo-replicates. Trees were visualized using ggtree (v 3.6.2) (Yu et al., 2018). **(C)** Principal component analysis of 434,890 SNP sites from the assessed 176 *S. cerevisiae* strains. Population differences indicated by color; NS, not specified. **(D)** Population structure of the 434,890 SNPs sites of the *S. cerevisiae* strains used in this study. Resolved population fractions are represented by the vertical axis; colors denote estimated ancestral membership. Varying the number of ancestral populations (K) between 1 and 10 using the simple prior implemented in fastSTRUCTURE (Raj et al., 2014), *K* = 8 was found to be optimal.

**Figure 2:**
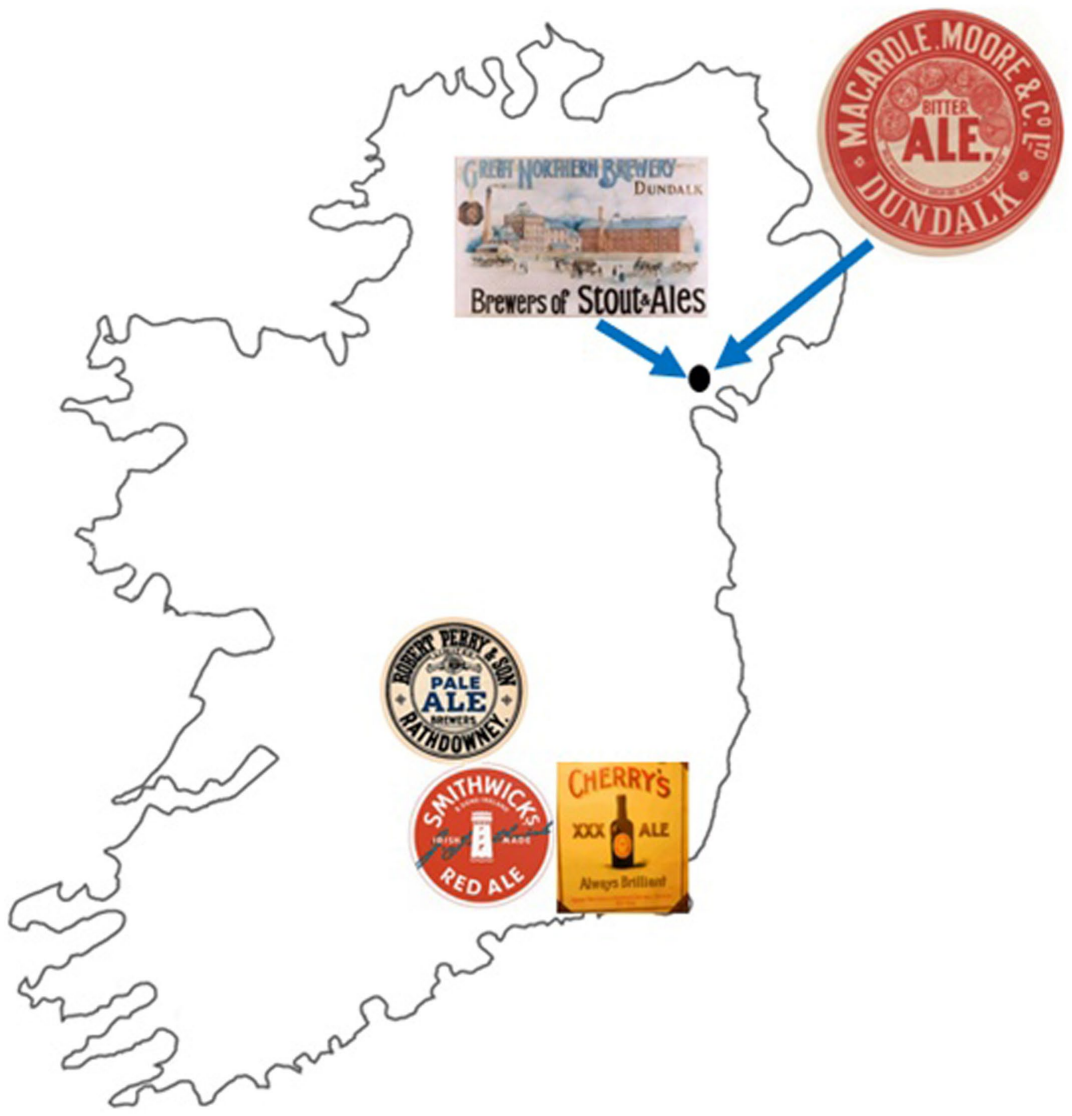
Location of historical Irish breweries. The Great Northern Brewery and Macardle Moore Brewery were located in the city of Dundalk, Ireland. The Smithwick’s brewery was located in the county of Kilkenny, while Cherry was within the county of Wexford and Perry within the county of Laois.

To understand the phylogeny of the six historical Irish brewing yeasts, a total of three colonies from Perry, Smithwick’s, Great Northern Brewery, Macardle 1965, Macardle 1993, and Cherry were selected. The resultant “fingerprints” (Supplementary Presentation 2) from the PCR analysis of the three colonies were assessed using hierarchical clustering (Euclidean distance) with identical yeast given a 100% similarity score. With the exception of Smithwick’s yeast, which had a similarity score of >98%, all three colonies of the other historical brewing yeasts had a similarity score <95% (Figure 1). The origins of the six historical Irish brewing yeasts were probed by comparing their genomes with those of previously published Irish brewing yeasts: 16 Guinness yeast and 154 previously published *S. cerevisiae* strains (Gallone et al., 2016; Kerruish et al., 2024). A total of 466,327 filtered variant sites were identified: 434,890 SNPs and 31,427 indels. For the historical Irish brewing yeasts, the number of transition and transversion SNPs, indels and singletons were determined (Table 2). Previous work established that the *S. cerevisiae* used to produce beer grouped within two lineages: Beer 1 and Beer 2 (Gallone et al., 2016). Our analysis confirmed the previous observations and placed the historical Irish brewing yeasts within the Beer 1 clade (Figure 1). To understand the origins of the historical Irish yeast populations, structure and degree of admixture were determined for the 176 genomes studied here using FastSTRUCTURE (Version 1.0) (Raj et al., 2014). The historical Irish brewing yeasts had a >80% common ancestry with yeast described as being in the “Britain” subpopulation (Gallone et al., 2016). The assessment of the historical Irish brewing yeasts established different phylogeny for the six brewing yeasts. The Perry, Cherry, and Smithwick’s yeasts showed 100% alignment with the “Britain” subpopulation; whereas, the Great Northern, Macardle 1966, and Macardle 1993 yeasts aligned with the “Britain” group but also the US and Belgium/Germany subpopulations. The SNPs of the Macardle 1965 yeasts shared a lineage of 2.91% Belgium/Germany, 89.13% Britain, and 7.96% United States. Macardle 1993 shared a lineage of 3.11% Belgium/Germany, 88.59% Britain, and 8.3% United States. The Great Northern Brewery shared a lineage of 4.91% Belgium/Germany, 87.36% Britain, and 7.74% United States. The phylogenetic tree (Figure 1) establishes that the six historical Irish brewing yeasts fall into two distinct groupings: the first group includes Macardle and Great Northern brewing yeasts, and the second group consists of Smithwick’s, Perry, and Cherry (Figure 2).

**Table 2:** Sporulation percentage; mean sequencing coverage along *S. cerevisiae* S288c genome, transition, transversion and singleton SNPs, and total indels of the six sequenced historical Irish brewing yeasts.

Yeast	Sporulation (%)	Average sequencing coverage (x)	Total transition SNPs	Total transversion SNPs	Number of singletons	Total indels
Cherry	0	417.6	59,787	20,606	142	7,033
Macardle 1965	1.09	391.8	59,740	20,579	168	7,075
Macardle 1993	0.13	713.4	59,841	20,659	30	7,099
Great Northern Brewery	0.03	1,230.1	59,226	20,494	56	7,111
Perry	0	770.5	58,795	20,238	39	7,017
Smithwick’s	0	767.8	60,012	20,731	1,676	7,198

Sporulation percentage was determined using the ASBC sporulation method (Bilinski and Casey, 1989). Sequencing analysis of the historical Irish brewing yeasts was performed using an Illumina MiSeq machine reading with a minimum depth of 30X coverage using 2 × 250 bp paired reads. SNPs and indels were determined through *de novo* assembly of the historical Irish brewing yeasts compared to the reference yeast *S. cerevisiae* S288c.

### Copy number variation and chromosomal arrangement of the historical Irish brewing yeasts

**Figure 3:**
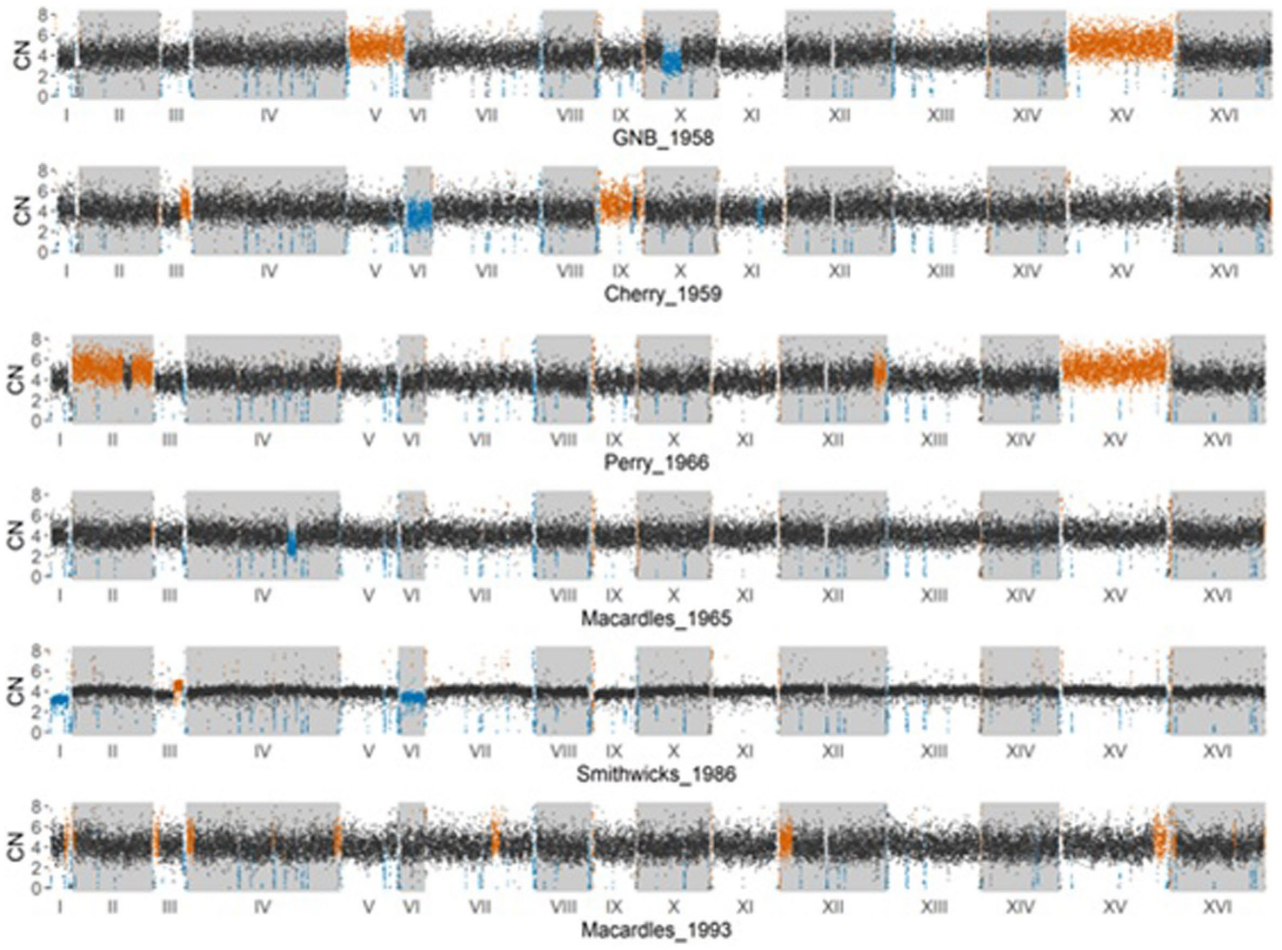
Estimated copy number variation in 250 base pair non-overlapping windows across the entire genome of the six historical Irish brewing yeasts. A black dot on a plot represents a window where the estimated copy number is 4. A blue dot represents a region with an estimated loss of copy number (<4), and an orange dot represents a region with an estimated increased copy number (>4).

To determine the copy number variation (CNV) in different Irish brewing yeasts, a full normalized read depth analysis was performed in 250-bp windows across each chromosome. The likely accuracy of the Irish brewing yeast ploidy estimates was confirmed by reassessing the ploidy of previously published yeast samples (Gallone et al., 2016). The 250-bp window assessment of the Irish brewing yeasts (Figure 3) showed the presence of multiple copies of chromosomes and CNVs of individual chromosomes. The Great Northern Brewery, Perry, and Cherry yeasts have chromosomes with more than four copies on chromosomes V, XV, and IX, respectively. Only Smithwick’s and Cherry have chromosomes with less than four copies on chromosomes I, VI, and VI.

All of the historical Irish brewing yeasts have multiple copies of their chromosomes and are, therefore, aneuploids. Aneuploidy is common for yeasts that are used in brewing (Boulton and Quain, 2008; Gallone et al., 2016; Morard et al., 2019) and is a consequence of the domestication of yeasts by human activity. Unlike natural isolates, which are likely to be diploid (Peter et al., 2018), brewing yeasts are aneuploid as selection pressures result in polyploidy for chromosomes conferring desirable phenotypic qualities (Voordeckers and Verstrepen, 2015). Diploid yeast strains have superior global cellular fitness than aneuploids (Peter et al., 2018), providing an advantage in changing environmental conditions and possessing a functional sexual phenotype (Morard et al., 2019). The sporulation ability of the Irish historical brewing yeasts (Table 2) confirms the effects of aneuploidy on a functional sexual phenotype, with Cherry, Perry, and Smithwick’s recording 0% sporulation, while Great Northern and Macardle brewing yeasts exhibited very low sporulation ability (<1.1%). This observation is concurrent with previous observations of aneuploidy and sporulation activity (Bilinski and Casey, 1989).

### Assessment of fermentation properties

**Figure 4:**
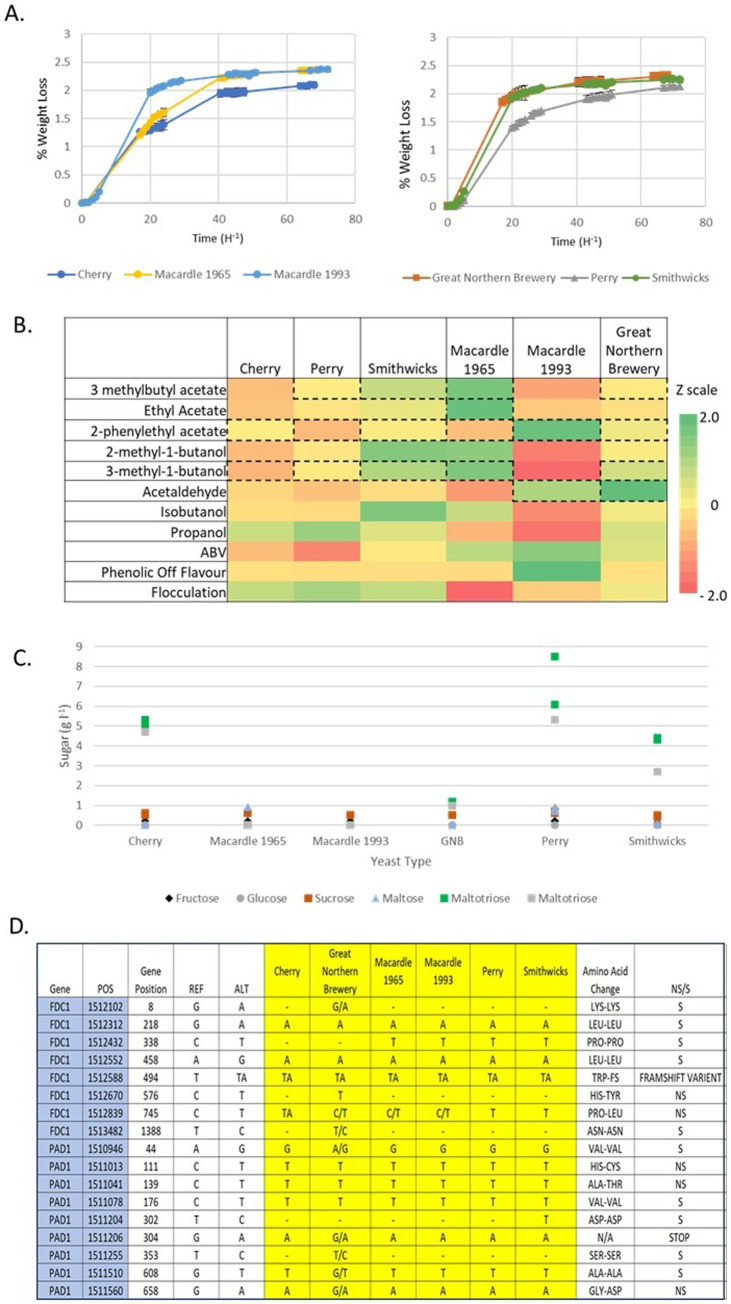
Phenotypic assessment of the historical Irish brewing yeast percentage weight loss **(A)**, phenotypic qualities represented through a heat map **(B)**, and sugar concentration at the end of fermentation **(C)**. Heat map Z-scores (normalized values) were used to determine flavor and phenotypic values, with the score color-coded according to the scale on the right. Flavor chemicals highlighted with a dotted line **(B)** are above the perceived flavor threshold. The single nucleotide polymorphism mutations of the POF genes *FDC1* and *PAD1* **(D)** of the different historical Irish brewing yeasts. The effects of the SNP mutations result in amino acid substitutions that are nonsynonymous (NS) or synonymous (S). The fermentations were performed using 100 mL of 12^o^P all malt wort, with an inoculation rate of 1 × 10^7^ ml^−1^ cells. Samples were incubated at 25°C and stirred at 250 rpm. Observations presented are *n* = 3 biologically independent experiments.

The fermentation properties of the historical Irish brewing yeasts were determined using the mini-fermentation procedure described in the methods section (Figure 4). A one-way ANOVA with Tukey’s *post hoc* test of ethanol concentration at the end of fermentation determined that ethanol production showed highly significant differences (*p* = 0.000376). However, when the ethanol concentration analysis was repeated based on 100% “British” SNP lineage (Perry, Cherry, and Smithwick’s) and <90% “British” lineage (Macardle 1960, Macardle 1993, and Great Northern Brewery), the *p*-values showed no significant difference (*p* = 0.12 and *p* = 0.09, respectively).

The progress of brewing fermentations is typically assessed by recording the decrease in wort density (attenuation), as this correlates with sugar utilization and ethanol formation. Measuring loss of weight, in the mini-fermentations described here, is an indirect method, which also relies on a fall in density. Under the conditions employed, all six historical Irish brewing yeasts achieved attenuation within 48 h with the Smithwick’s yeast completing attenuation within 30 h. Brewing yeast strains can assimilate simple wort sugars, which include glucose, fructose, sucrose, maltose, and maltotriose; dextrins are not fermented (Briggs et al., 2004). Analysis of the sugar spectrum at the end of fermentation (Figure 4; Supplementary Table 1) established that all historical Irish brewing yeasts utilized the typical brewing sugars found in wort.

There was no statistical significance between the yeasts for glucose and fructose utilization, as all the brewing yeasts utilized these available sugars (*p* = 0.17, one-way ANOVA). A one-way ANOVA of sucrose utilization (*p* = 0.0075) established that there were statistically different usages between all tested yeasts; however, when analyzed based on regional location, differences were observed for the Cherry, Perry, and Smithwick’s (*p* = 0.014), but not when Cherry and Perry yeasts were compared alone (*t*-test 0.23). There were no statistical differences in sucrose utilization between Macardle and Great Northern Brewery yeasts (*p* = 0.125). Maltose was fully utilized by all the yeasts, except for the Perry, Smithwick’s, and Macardle 1965 yeasts; whereas, maltotriose was only utilized fully by the two Macardle yeasts. A one-way ANOVA of maltotriose utilization of Cherry, Perry, and Smithwick’s was not statistically significant (*p* = 0.056) but was when Cherry, Perry, Smithwick’s, and Great Northern Brewery were compared (*p* = 0.00078).

### Gene function

Five maltotriose utilization multigene loci: *MAL 1*, *2*, *3*, *4*, and *6* have been identified in *S. cerevisiae* (Charron et al., 1989). The *MAL* locus comprises three genes: a maltose permease (gene 1), maltase (gene 2), and a *Trans* acting *MAL*-activator (gene 3) (Charron et al., 1989). Of the five multigene loci, only *MAL1* and *MAL3* multigenes are present within the reference yeast *S. cerevisiae* S288c (Gallone et al., 2016). Assessment of *MAL1* and *MAL3* (Supplementary Table 2) established a homozygous premature stop codon in the *MAL11* maltose permease gene for all six historical brewing yeasts. This stop codon mutation, which was found in 145 of the 176 yeasts examined in this study, potentially prevents the loss of gene function.

Assessment of the *MAL31* maltose permease gene established that the Perry, Cherry, and Great Northern Brewery yeasts contain no SNPs different from the reference yeast S288c, whereas the Smithwick’s yeasts contain two heterozygous SNPs at positions 803,680 and 803,689–803,693. All six historic brewing yeasts utilized maltose and maltotriose, but only the Macardle yeasts fully utilized maltotriose. Comparison of the Macardle yeasts to S288c established that there are 15 SNPs: 13 heterozygous and 2 homozygous. The homozygous SNPs at positions 802,839 and 802,842 increased the number of DNA bases within the *MAL31* open reading frame (ORF). Further experimental investigation is required to determine whether the mutations in *MAL31* are responsible for the increased maltotriose utilization observed in the Macardle yeasts or whether other *MAL* multigene loci are present.

### Production of flavor metabolites by historical Irish brewing yeasts

The concentrations of flavor-active esters (three methylbutyl acetate [isoamyl acetate], 2-phenylethyl acetate, and ethyl acetate), amyl alcohols (2-methyl-1-butanol and 3-methyl-1-butanol), and the aldehyde acetaldehyde were measured at the cessation of fermentation (Figure 4). The concentration of 2-phenylethyl acetate was below the flavor threshold (3.8 ppm) for all beers made using the historical Irish brewing yeasts. Perry, Smithwick’s, Great Northern, and Macardle 1965 yeasts all produced Isoamyl acetate above the flavor threshold of 1.1 ppm (Meilgaard et al., 1982). The Macardle 1993 and Cherry yeast generated lower concentrations of three methylbutyl acetate (isoamyl acetate): 0.71 ppm (SD 0.31) and 1.07 ppm (SD 0.11), respectively. All historical Irish brewing yeasts produced ethyl acetate at concentrations below the 33-ppm flavor threshold (Meilgaard et al., 1982), with the exception of Macardle 1965. A one-way ANOVA of flavor esters established that all historical yeasts produced statistically significant amounts of esters: isoamyl acetate (*p* = 2.77 × 10^−5^), 2-phenylethyl acetate (*p* = 7.80 × 10^−6^), and ethyl acetate (*p* = 0.00043).

Concentrations of 2-methyl-1-butanol were below the flavor threshold of 65 ppm (Meilgaard et al., 1982), contrary to 3-methyl-1-butanol, which was above the flavor threshold of 70 ppm (Meilgaard et al., 1982), in all historical Irish brewing yeasts except the Macardle 1993 yeast. Acetaldehyde was found to be above the flavor threshold (10 ppm, Meilgaard et al., 1982) for the Macardle 1993 and Great Northern Brewery yeasts only. All other historical Irish brewing yeasts were below the 10-ppm flavor threshold. Unlike other phenotypic attributes examined in this study, the ester and amyl alcohol concentrations did not correlate with the 100% “British” SNP and < 90% “British” lineages (Supplementary Table 1), except 2-phenylethyl acetate and acetaldehyde that had a *p*-value of 0.182 and 0.788 (one-way ANOVA), respectively, for the 100% “British” SNP lineage.

Propanol and isobutanol are higher alcohols (Fusel alcohols) produced by the yeast via the Ehrlich pathway (Briggs et al., 2004). A one-way ANOVA of propanol production by the historical Irish brewing yeasts (Figure 4) established that propanol production was statistically significantly different between the historical Irish brewing yeasts (*p* = 0.000029). However, when propanol production was assessed by a regional grouping of 100% “British” SNP lineage and <90% “British” lineage, the *p*-values were 0.11 and 0.00072, respectively, with propanol production being statistically significant in the <90% British lineage yeasts. This observation is contrary to isobutanol production among the historic Irish brewing yeasts, where a one-way ANOVA established statistically significant differences (*p* = 6.048 × 10^−9^) when assessed by the 100% “British” SNP lineage and < 90% “British” lineage (*p* = 7.36 × 10^−5^ and *p* = 1.23 × 10^−6^).

The Ehrlich pathway produces higher alcohols via a process of transamination, decarboxylation, and reduction (Lilly et al., 2006). Assessment of the CNV of the Ehrlich pathway (Supplementary Table 3) established that all historical Irish brewing yeasts had four copies of the *BAT2* gene. Overexpression of the aminotransferase-encoded *BAT2* gene has been proven to increase alcohol production (Lilly et al., 2006; Styger et al., 2013). However, this study establishes that the detected differences in higher alcohol production among the historical Irish brewing yeasts are not a consequence of increased CNV of *BAT2*. Furthermore, the absence of a CNV effect on higher alcohol production is supported by consideration of CNV in the oxidation genes *ADH1* for the Great Northern Brewery and Perry yeasts. Both of these have five copies of the *ADH1* gene compared to four in the other historical Irish brewing yeasts, yet they do not produce the highest concentration of isobutanol. This was observed in the Smithwick’s yeast (72.18 ppm, SD 3.32). In the case of propanol production, Perry’s yeast produced 36.01 ppm (SD 1.18), the highest concentration of all the historical Irish yeasts assessed in this study. CNV differences within the historical Irish brewing yeasts for genes involved in the reduction stage of the Ehrlich pathway were not assessed in this study, so the potential impact of this feature within the Irish brewing yeast group remains unknown.

### POF production by historical Irish brewing yeast

Phenolic off-flavor (POF) imparts a medicinal, clove-like flavor in beer (Madigan et al., 1994). This is due to 4-vinyl-guaiacol produced from the precursor ferulic acid via the expression of the genes *PAD1* and *FDC1* (Mukai et al., 2014) (Figure 4). These genes encode a phenylacrylic acid decarboxylase (*PAD1*) and a ferulic acid decarboxylase (*FDC1*), which enable the yeast to decarboxylate the phenylacrylic acid ferulic acid (Mukai et al., 2014). Ferulic acid is present in cereals and is released during the mashing step in wort production (Madigan et al., 1994). For beers brewed in the Hefeweizen style, the presence of 4-vinyl-guaiacol at concentrations higher than the threshold value is desirable, so the mashing process is controlled to favor the adequate formation of ferulic acid (Briggs et al., 2004). For the majority of beers, the presence of detectable concentrations of 4-vinyl-guaiacol is considered a negative flavor attribute; consequently, POF-negative yeasts are often selected (Gallone et al., 2016). Only the Macardle 1993 yeast produced 4-vinyl-guaiacol above the beer flavor threshold (200–400 ppb) (Madigan et al., 1994). The presence of four vinyl-guaiacol of the other historical Irish brewing yeasts—Perry, Cherry, Smithwick’s, Great Northern Brewery, and Macardle 1965 was below the flavor threshold; subsequently, these yeasts would be termed as being POF-negative. Assessment of the *PAD1* and *FDC1* genes confirms these observations, as all five POF-negative historical Irish brewing yeasts contain deleterious mutations in the *PAD1* and *FDC1* genes (Figure 4). Furthermore, all yeasts exhibited a loss of heterozygosity (Figure 4). Only the Great Northern Brewery yeast retained a majority of heterozygous SNPs with the Cherry, Perry, and Smithwick’s yeast losing heterozygosity in all different SNPs compared to the reference yeast *S. cerevisiae* S288c. A mutation at position 494 in *FDC1* results in a frameshift mutation, and a stop mutation at position 304 in *PAD1* has previously been described as leading to a POF-negative phenotype (Gonçalves et al., 2016). The data presented in this study confirmed that Perry, Cherry, Smithwick’s, Great Northern Brewery, and Macardle 1965 were all POF-negative. All historical Irish brewing yeasts have a homozygous SNP at position 304 in the *PAD1* gene, except for the Great Northern Brewery yeast that retains a heterozygous SNP. This SNP at position 304 results in a stop codon in *PAD1*. The Great Northern Brewery is POF-negative establishing that the heterozygous mutation at position 304 in *PAD1* does not result in retention of the POF phenotype.

The Macardle 1993 yeast retains both the frameshift mutation in *FDC1* and the stop codon mutation in *PAD1*. Nevertheless, the Macardle 1993 yeast is POF-positive producing 4-vinyl-guaiacol at a concentration >1,000 ppm. A repeated sniff test confirmed the original observation that the Macardle 1993 yeast is POF-positive. The observation that a yeast can be POF-positive despite having an adenine insertion in *FDC1* and the presence of a stop codon in *PAD1* has been previously reported in a British yeast that grouped within the Hefeweizen yeast cluster (Gonçalves et al., 2016). However, these mutations to *FDC1* and *PAD1* would be expected to result in a loss of function (Mukai et al., 2014).

### Flocculation phenotype of historical Irish brewing yeast

The yeast flocculation phenotype is exploited by brewers as an aid to beer clarification (Vidgren and Londesborough, 2011). This reversible, non-sexual aggregation of cells improves the efficiency of sedimentation or separation from beer at the end of fermentation. This aids both beer colloidal stability and the ease of yeast crop separation (Vidgren and Londesborough, 2011). Environmental factors, such as pH, temperature, Ca^2+^ concentration, fermentable sugars, and other nutrients, influence the efficiency of flocculation (Stratford, 1989; Stratford, 1992; Straver et al., 1993; Stratford, 1996). The flocculation genes *FLO1, FLO5, FLO8, FLO9, FLO10*, and *FLO11* are responsible for determining the flocculation phenotype (Govender et al., 2008). Using the EBC Gilliland Method EBC 3.5.31, the flocculation of the six historical Irish brewing yeast was determined (Gilliland, 1951) (Figure 4). Gilliland’s method places yeast flocculation within defined flocculation classes: Class 1, non-flocculant; Class 2, slightly flocculant; Class 3, moderately flocculant; Class 4, highly flocculant. The 100% “British” yeasts were all Class 4, highly flocculant. The Macardle 1965 yeast was Class 2, slightly flocculant, while the Macardle 1993 and Great Northern Brewery yeasts were moderately flocculant. Further analysis of the effects of the flocculation genes *FLO1*, *FLO5*, *FLO8*, *FLO9*, *FLO10*, and *FLO11* was not possible with the Illumina sequencing data, as these genes are located in the subtelomeric regions: areas associated with poor quality genome reads (Teunissen and Steensma, 1995).

The historical records of the flocculation phenotypes of the six Irish yeasts were different from the observations of this study. The 100% “British” lineage yeasts were all reported as being Class 2, slightly flocculant; whereas, the Macardle 1965 yeast was Class 3, moderately flocculant, the Macardle 1993 yeast was Class 2, slightly flocculant, and the Great Northern Brewery yeast was Class 4, highly flocculant. The EBC Gilliland Method EBC 3.5.31 is subjective, as it requires the user to determine the level of flocculence based on a set of flocculation criteria. This may account for a difference in the historical and recent observations, or perhaps there was a change occurred in flocculation phenotype, although the loss of flocculation ability is the more common mutation (Sato et al., 2001). The assessment of flocculation phenotype established that there were no observable flocculation phenotype differences among the 100% “British” lineage yeasts, but differences were observed among the <90% “British” lineage yeasts.

## Discussion

The results presented in this study support the contention that the historical Irish brewing yeasts examined form distinct phylogenetic groups that correlate with specific regional locations in Ireland, specifically those from the contiguous southern counties of Laois, Kilkenny, and Wexford grouping, and the more northerly Dundalk yeast. Furthermore, brewing-associated phenotypes also group within these regional locations. Previous publications established that the *Saccharomyces* genus is of Asian origin (Peris et al., 2023) with brewing strains of *S. cerevisiae*, an admixture of Asian and European wine strains (Fay et al., 2019). *S. cerevisiae* yeasts used to brew commercial beers have been found to be dominated by Asian admixture (Abou Saada et al., 2022), but the data presented in this study provide evidence of more recent admixture events. The data confirm that while the historical Irish brewing yeasts share a common ancestor, they have subsequently diverged genotypically and phenotypically to be associated with two distinct regional locations.

The historical Irish brewing yeast: Perry, Smithwick’s, Great Northern Brewery, Macardle 1965, Macardle 1993, and Cherry belong to the Britain lineage grouping, within the previously described Beer 1 clade (Gallone et al., 2016). A subset of segregating SNPs, generated by pruning based on high levels of pairwise linkage disequilibrium between variants, was used to determine the degree of shared ancestry between the historical Irish brewing yeasts and other previously sequenced, functionally and geographically variable samples. This population structure analysis confirms the placement of the historical Irish brewing yeasts within the geographically distinct Britain subgroup of the Beer 1 clade. It suggests that Cherry, Perry and Smithwick’s exhibit a 100% fractional representation of the resolved “British” lineage, whereas the Great Northern Brewery, Macardle 1965, and Macardle 1993 yeasts exhibit <90% “British” lineage, with the remaining variation sharing ancestry with the US subpopulation in the Beer 1 clade. The yeasts associated with a 100% “Britain” lineage are from counties that border one other—Laois, Kilkenny, and Wexford—whereas those with <90% “Britain” lineage are from the geographically distinct town of Dundalk.

In addition to their shared genetic 100% “British” ancestry, the Perry, Cherry, and Smithwick’s yeasts share phenotypic similarities, including patterns of glucose, fructose, and maltotriose utilization, flocculation characteristics, and the formation of ethanol, propanol, and POF. These are shared with the 100% “British” lineage yeasts. These are in contrast with the <90% “Britain” lineage where only ethanol and the sugars glucose, fructose, and sucrose utilization are similar. When all historical Irish brewing yeasts are assessed for phenotypic similarity, only glucose and fructose utilization were not statistically significant. This is unsurprising, as glucose and fructose are the sugars used as the starting point for glycolysis (Briggs et al., 2004).

All yeasts assessed in this study are aneuploid with multiple CNVs. The role of CNV on phenotype is an area of study where there is conflicting information. For example, publications have demonstrated the effects of CNV on adaptation to toxic chemical stress (Liu and Huang, 2022), improved resistance to acidic conditions (Kang et al., 2019), and improved ethanol tolerance (Morard et al., 2019). However, in this study, the assessment of CNV of genes related to brewing phenotypes for the historical Irish brewing yeasts confirms that CNV does not increase or decrease brewing-related phenotypes. This observation confirms previous studies where CNV does not affect brewing phenotypes (Scopel et al., 2021).

POF is undesirable in beers that are not brewed in the Hefeweizen style (Gallone et al., 2016). The loss of the POF phenotype is commonplace for yeasts used to make commercial beer and sake (Gallone et al., 2016). All six historical Irish brewing yeasts contain mutations in the *PAD1* and *FDC1* genes that have been reported as negatively impacting the POF phenotype (Mukai et al., 2014; Chen et al., 2015). The phenotypic assessment of POF confirms the genotype in five of the six historical Irish brewing yeasts: Perry, Smithwick’s, Great Northern Brewery, Macardle 1965, and Cherry; however, despite containing the same mutations that result in a loss of function, the Macardle 1993 yeast is POF-positive. Previous publications have hypothesized that as yet unidentified compounds or enzymes result in a change in POF production (Gonçalves et al., 2016; Kerruish et al., 2024). The data presented in this study further support these observations, as the reported insertion in *FDC1* and the presence of a stop codon in *PAD1* has been previously reported in a British ale POF-positive yeast, TUM 507 (Gonçalves et al., 2016). The data presented in this study confirm that the observation in TUM 507 occurs in another brewing yeast and warrants further investigation.

The historical records of the yeast used in this project provide further context to the genotypic and phenotypic observations of this study. The extant historical records held within the Guinness archives and the yeast library at St. James’s Gate give insight as to which yeast was used in which brewery. For example, the Cherry yeast used in this study was the original yeast used in the Cherry New Ross Brewery, even though later Cherry beers were brewed in the Strangman Brewery. However, the observations of Macardle yeast are incongruous with the historical records in which it states that the Macardle 1993 pitching yeast was isolated from the 1970 Smithwick’s brewing pitching yeast. The data presented in this study establish that both of the Macardle yeasts share a recent common ancestor that is removed from the Smithwick’s yeast. Moreover, the 1970 Smithwick’s pitching yeast was POF-negative whereas, the Macardle 1993 yeast is POF-positive. Perhaps the reason for the difference in the historical context and the observation of this study is that, in 1993, Smithwick’s was brewed in the Macardle brewery. Consequently, the yeast isolated from Macardle in 1993 could have been the Macardle brewery house yeast. The genotypic analysis of the Macardle yeast supports this hypothesis.

All yeasts assessed in this study produced a mixture of ales and stouts (Colgan, 2020). In addition, the Cherry, Perry, and Smithwick’s breweries produced pale ales, India Pale ales, porters, and barley wines (Colgan, 2020). In our previous publication, we hypothesized that the reason for the difference between the Guinness yeast and other historical Irish brewing yeasts was that Guinness yeast was used to brew beer principally in the stout style only (Kerruish et al., 2024). As the Guinness yeast was used for stout production, it meant that phenotypic traits that would usually be undesirable, such as poor flocculation and POF-positive, could be retained as they did not have a negative impact on stout production (Kerruish et al., 2024). Consequently, for brewers producing different beer styles, a yeast that was generalist would be the most suitable. The data presented in this study add further support to this hypothesis. The 100% “British” lineage yeast—Perry, Cherry, and Smithwick’s—were used to produce a range of different beer styles and share phenotypic traits, including the same flocculation class, POF-negative, and patterns of utilization of glucose, fructose, and maltotriose. These phenotypes would be desirable in a yeast used to brew different beer styles, a generalist yeast. The phenotypic traits that are statistically different among Perry, Cherry, and Smithwick’s are principally phenotypes related to flavor, a personal choice of the brewer as opposed to a requirement of brewing. In contrast, the <90% “British” lineage—Great Northern Brewery, Macardle 1965, Macardle 1993 yeast—produced ales with limited amounts of stouts; consequently, these yeasts share fewer phenotypic traits. When all brewing phenotypes of the historical Irish brewing yeasts are compared, they are statistically different. However, when the phenotypes are assessed based on 100 and <90% “Britain” lineage, differences in phenotypes are limited. Accordingly, like the genotypic grouping, the phenotype grouping is divided upon 100 and <90% “Britain” lineage.

The six historical yeasts group phenotypically and genotypically within the requirements of their beer-style production. The generalist yeasts, 100% “Britain” lineage, were used to brew different beer styles from Indian pale ales to low Alcohol By Volume ales, whereas the <90% “Britain” lineage brewed beer in principally one beer style (Colgan, 2020). Previous SNP assessment of *S. cerevisiae* by Gonçalves et al. (2016) placed yeast within beer styles; however, there were notable differences with some yeasts determined as being from a particular beer-style grouping outside of their stated category. Similarly, other microbes used in the manufacturing process are also grouped by their industrial application. Phylogenetic assessment using multi-locus sequencing typing of the milk-processing lactic acid bacteria *Lactococcus lactis* grouped the bacterial species within the dairy and non-dairy-associated strains (Passerini et al., 2010). The grouping established that cheese production associated with *L. lactis* groups closely with starting culture *L. lactis* and that these groupings are different from the non-domesticated *L. lactis.* Similarly, the phylogenetic assessment of industrial *A. oryzae* was grouped based on industrial applications (Watarai et al., 2019). Furthermore, the phylogenetic assessment of 50 *Oenococcus oeni* strains, a lactic acid bacteria associated with malolactic fermentation of wine and cider production, grouped within their respective product type. The authors concluded that these groupings were a consequence of domestication activity (Campbell-Sills et al., 2015). These studies of microbes used by humans in the manufacturing process established the phylogenetic relationship between microbes, demonstrating the effects of domestication on their respective genomes. The data presented in this study provide further evidence of the effects of domestication, with the phenotype of the yeast relating to brewing functionality. Our data provide further evidence of domestication influencing microbial phenotype and genotype, establishing that there are specific yeast for specific beer styles. Moreover, for breweries that make a particular beer, this study suggests that the yeast strain is specific to that beer. This observation has potentially wider implications for microbe-derived food and beverage products establishing the importance of proprietary microbes for specific products.

The phylogenetic data presented in this study establish that the six historical Irish brewing yeasts from five Irish breweries are divided into two lineages: 100% “Britain” and <90% “Britain” and that brewing phenotypes of these yeasts also group within these lineages. Our findings establish that the brewing phenotypes observed within the different yeasts are a consequence of the different beer styles brewed with “generalist” brewing yeasts sharing brewing phenotypes that enable the production of a range of different beers, whereas yeast used to make stouts and ales have specific brewing phenotypic traits. Furthermore, the CNV analysis of key brewing phenotypes confirms previous observations that CNV does not influence brewing phenotypes. Moreover, the analysis presented in this study adds further evidence that there are as yet unidentified genes within *S. cerevisiae* that produce POF, as mutations in the POF genes *PAD1* and *FDC1* still result in retention of the POF phenotype. Finally, in conclusion, this study provides further evidence of yeast selection influencing beer styles.

## Data Availability

Illumina and Nanopore (basecalled, demultiplexed) reads for all sequenced samples in this manuscript are deposited in the European Nucleotide Archive (ENA) under the project accession PRJEB62101. All experimental data is presented in Supplementary Table 1.
