# Supplementary material for: Effects of regional location on the genotype and phenotype of historical Irish brewing yeast
Source: Front Microbiol. 2025 Mar 11;16:1452334. doi: 10.3389/fmicb.2025.1452334 (PMC11933050; doi:10.3389/fmicb.2025.1452334)
Supplement: Supplementary file 4 [file Presentation_1.pptx]

## Slide 1
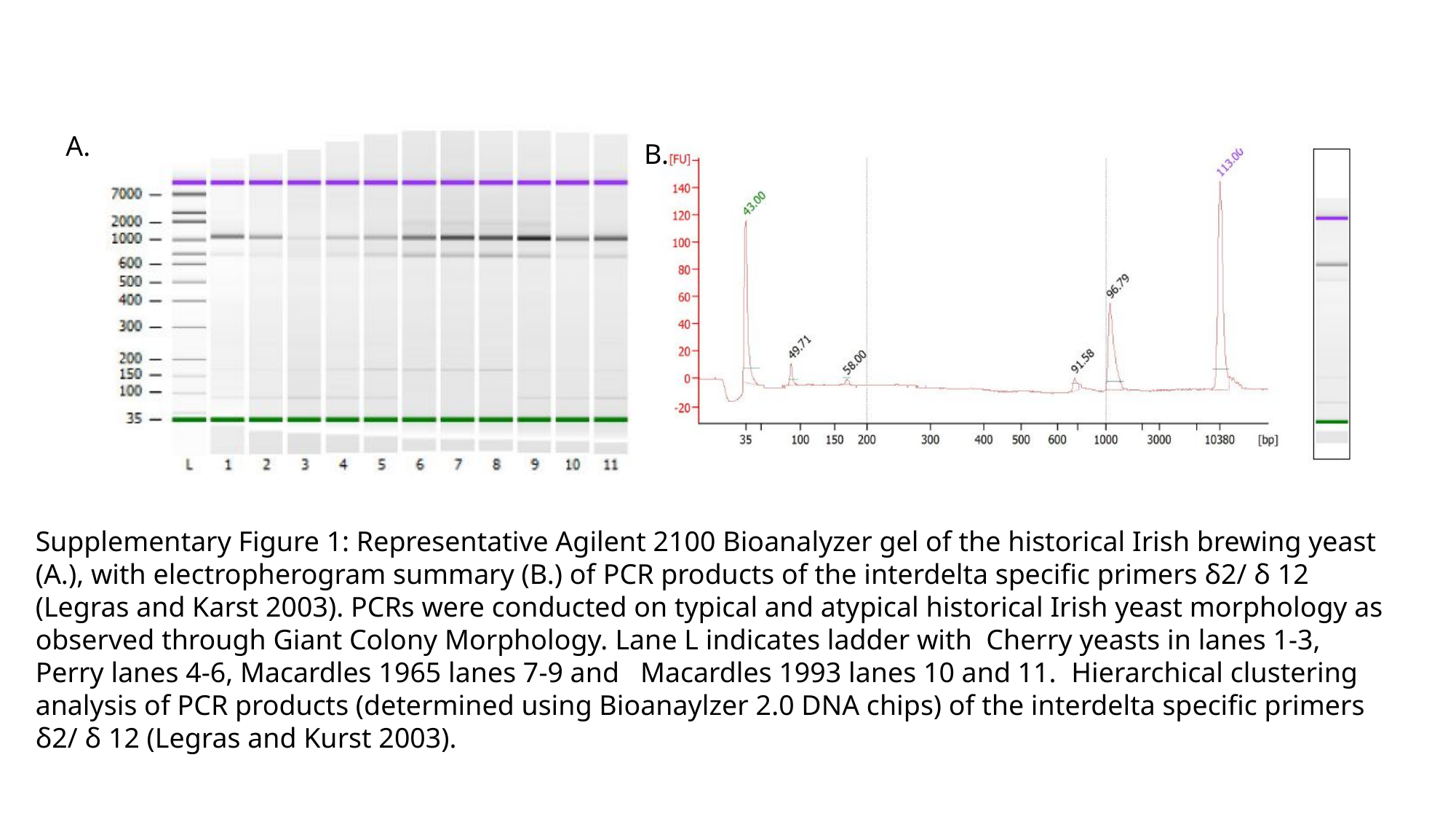

A.
B.
Supplementary Figure 1: Representative Agilent 2100 Bioanalyzer gel of the historical Irish brewing yeast (A.), with electropherogram summary (B.) of PCR products of the interdelta specific primers δ2/ δ 12 (Legras and Karst 2003). PCRs were conducted on typical and atypical historical Irish yeast morphology as observed through Giant Colony Morphology. Lane L indicates ladder with Cherry yeasts in lanes 1-3, Perry lanes 4-6, Macardles 1965 lanes 7-9 and Macardles 1993 lanes 10 and 11. Hierarchical clustering analysis of PCR products (determined using Bioanaylzer 2.0 DNA chips) of the interdelta specific primers δ2/ δ 12 (Legras and Kurst 2003).
