# Supplementary material for: Effects of regional location on the genotype and phenotype of historical Irish brewing yeast
Source: Front Microbiol. 2025 Mar 11;16:1452334. doi: 10.3389/fmicb.2025.1452334 (PMC11933050; doi:10.3389/fmicb.2025.1452334)
Supplement: Supplementary file 5 [file Presentation_2.pptx]

## Slide 1
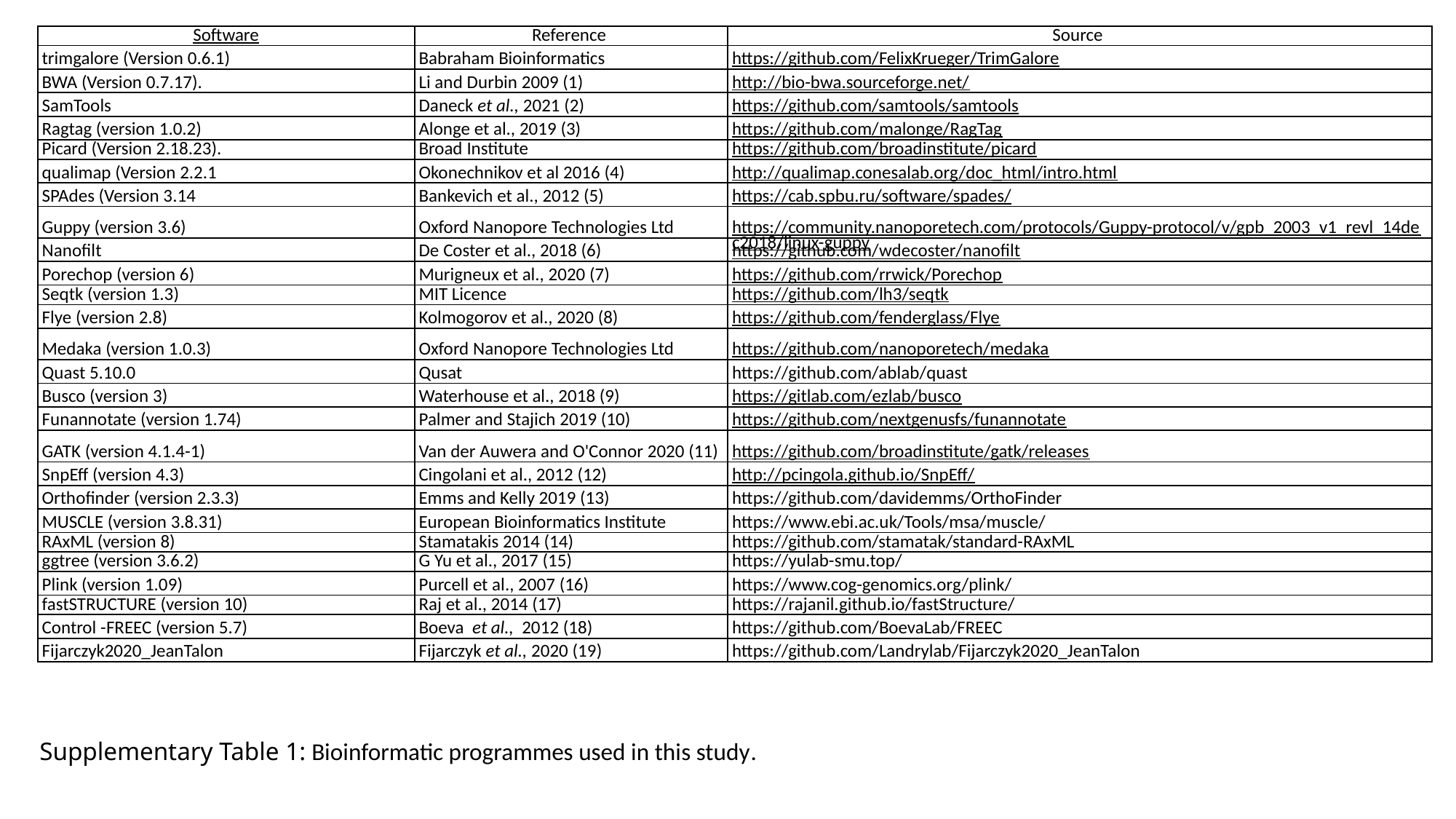

| Software | Reference | Source |
| --- | --- | --- |
| trimgalore (Version 0.6.1) | Babraham Bioinformatics | https://github.com/FelixKrueger/TrimGalore |
| BWA (Version 0.7.17). | Li and Durbin 2009 (1) | http://bio-bwa.sourceforge.net/ |
| SamTools | Daneck et al., 2021 (2) | https://github.com/samtools/samtools |
| Ragtag (version 1.0.2) | Alonge et al., 2019 (3) | https://github.com/malonge/RagTag |
| Picard (Version 2.18.23). | Broad Institute | https://github.com/broadinstitute/picard |
| qualimap (Version 2.2.1 | Okonechnikov et al 2016 (4) | http://qualimap.conesalab.org/doc\_html/intro.html |
| SPAdes (Version 3.14 | Bankevich et al., 2012 (5) | https://cab.spbu.ru/software/spades/ |
| Guppy (version 3.6) | Oxford Nanopore Technologies Ltd | https://community.nanoporetech.com/protocols/Guppy-protocol/v/gpb\_2003\_v1\_revl\_14dec2018/linux-guppy |
| Nanofilt | De Coster et al., 2018 (6) | https://github.com/wdecoster/nanofilt |
| Porechop (version 6) | Murigneux et al., 2020 (7) | https://github.com/rrwick/Porechop |
| Seqtk (version 1.3) | MIT Licence | https://github.com/lh3/seqtk |
| Flye (version 2.8) | Kolmogorov et al., 2020 (8) | https://github.com/fenderglass/Flye |
| Medaka (version 1.0.3) | Oxford Nanopore Technologies Ltd | https://github.com/nanoporetech/medaka |
| Quast 5.10.0 | Qusat | https://github.com/ablab/quast |
| Busco (version 3) | Waterhouse et al., 2018 (9) | https://gitlab.com/ezlab/busco |
| Funannotate (version 1.74) | Palmer and Stajich 2019 (10) | https://github.com/nextgenusfs/funannotate |
| GATK (version 4.1.4-1) | Van der Auwera and O'Connor 2020 (11) | https://github.com/broadinstitute/gatk/releases |
| SnpEff (version 4.3) | Cingolani et al., 2012 (12) | http://pcingola.github.io/SnpEff/ |
| Orthofinder (version 2.3.3) | Emms and Kelly 2019 (13) | https://github.com/davidemms/OrthoFinder |
| MUSCLE (version 3.8.31) | European Bioinformatics Institute | https://www.ebi.ac.uk/Tools/msa/muscle/ |
| RAxML (version 8) | Stamatakis 2014 (14) | https://github.com/stamatak/standard-RAxML |
| ggtree (version 3.6.2) | G Yu et al., 2017 (15) | https://yulab-smu.top/ |
| Plink (version 1.09) | Purcell et al., 2007 (16) | https://www.cog-genomics.org/plink/ |
| fastSTRUCTURE (version 10) | Raj et al., 2014 (17) | https://rajanil.github.io/fastStructure/ |
| Control -FREEC (version 5.7) | Boeva et al., 2012 (18) | https://github.com/BoevaLab/FREEC |
| Fijarczyk2020\_JeanTalon | Fijarczyk et al., 2020 (19) | https://github.com/Landrylab/Fijarczyk2020\_JeanTalon |
Supplementary Table 1: Bioinformatic programmes used in this study.

## Slide 2
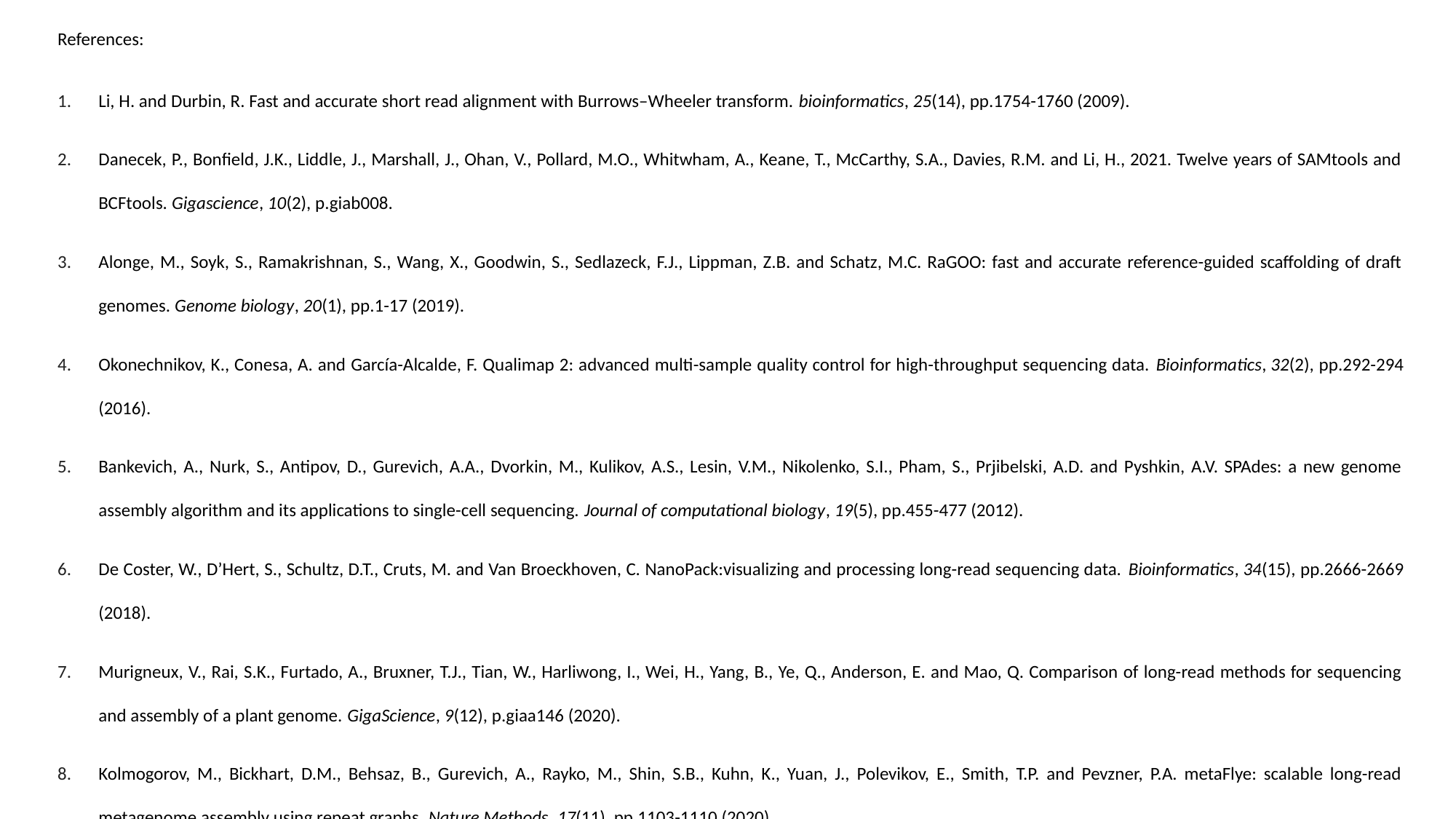

References:
Li, H. and Durbin, R. Fast and accurate short read alignment with Burrows–Wheeler transform. bioinformatics, 25(14), pp.1754-1760 (2009).
Danecek, P., Bonfield, J.K., Liddle, J., Marshall, J., Ohan, V., Pollard, M.O., Whitwham, A., Keane, T., McCarthy, S.A., Davies, R.M. and Li, H., 2021. Twelve years of SAMtools and BCFtools. Gigascience, 10(2), p.giab008.
Alonge, M., Soyk, S., Ramakrishnan, S., Wang, X., Goodwin, S., Sedlazeck, F.J., Lippman, Z.B. and Schatz, M.C. RaGOO: fast and accurate reference-guided scaffolding of draft genomes. Genome biology, 20(1), pp.1-17 (2019).
Okonechnikov, K., Conesa, A. and García-Alcalde, F. Qualimap 2: advanced multi-sample quality control for high-throughput sequencing data. Bioinformatics, 32(2), pp.292-294 (2016).
Bankevich, A., Nurk, S., Antipov, D., Gurevich, A.A., Dvorkin, M., Kulikov, A.S., Lesin, V.M., Nikolenko, S.I., Pham, S., Prjibelski, A.D. and Pyshkin, A.V. SPAdes: a new genome assembly algorithm and its applications to single-cell sequencing. Journal of computational biology, 19(5), pp.455-477 (2012).
De Coster, W., D’Hert, S., Schultz, D.T., Cruts, M. and Van Broeckhoven, C. NanoPack:visualizing and processing long-read sequencing data. Bioinformatics, 34(15), pp.2666-2669 (2018).
Murigneux, V., Rai, S.K., Furtado, A., Bruxner, T.J., Tian, W., Harliwong, I., Wei, H., Yang, B., Ye, Q., Anderson, E. and Mao, Q. Comparison of long-read methods for sequencing and assembly of a plant genome. GigaScience, 9(12), p.giaa146 (2020).
Kolmogorov, M., Bickhart, D.M., Behsaz, B., Gurevich, A., Rayko, M., Shin, S.B., Kuhn, K., Yuan, J., Polevikov, E., Smith, T.P. and Pevzner, P.A. metaFlye: scalable long-read metagenome assembly using repeat graphs. Nature Methods, 17(11), pp.1103-1110 (2020).

## Slide 3
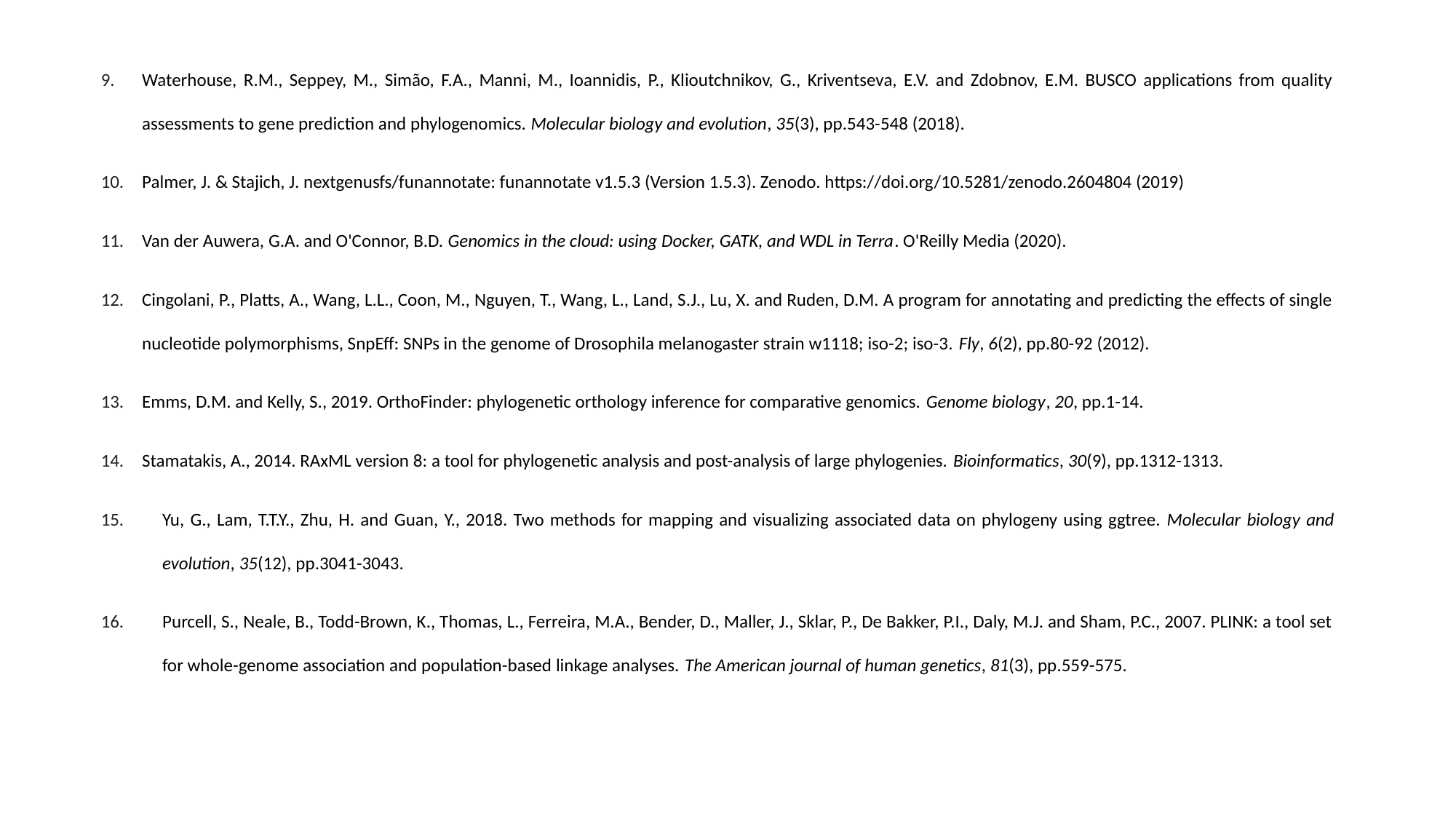

Waterhouse, R.M., Seppey, M., Simão, F.A., Manni, M., Ioannidis, P., Klioutchnikov, G., Kriventseva, E.V. and Zdobnov, E.M. BUSCO applications from quality assessments to gene prediction and phylogenomics. Molecular biology and evolution, 35(3), pp.543-548 (2018).
Palmer, J. & Stajich, J. nextgenusfs/funannotate: funannotate v1.5.3 (Version 1.5.3). Zenodo. https://doi.org/10.5281/zenodo.2604804 (2019)
Van der Auwera, G.A. and O'Connor, B.D. Genomics in the cloud: using Docker, GATK, and WDL in Terra. O'Reilly Media (2020).
Cingolani, P., Platts, A., Wang, L.L., Coon, M., Nguyen, T., Wang, L., Land, S.J., Lu, X. and Ruden, D.M. A program for annotating and predicting the effects of single nucleotide polymorphisms, SnpEff: SNPs in the genome of Drosophila melanogaster strain w1118; iso-2; iso-3. Fly, 6(2), pp.80-92 (2012).
Emms, D.M. and Kelly, S., 2019. OrthoFinder: phylogenetic orthology inference for comparative genomics. Genome biology, 20, pp.1-14.
Stamatakis, A., 2014. RAxML version 8: a tool for phylogenetic analysis and post-analysis of large phylogenies. Bioinformatics, 30(9), pp.1312-1313.
Yu, G., Lam, T.T.Y., Zhu, H. and Guan, Y., 2018. Two methods for mapping and visualizing associated data on phylogeny using ggtree. Molecular biology and evolution, 35(12), pp.3041-3043.
Purcell, S., Neale, B., Todd-Brown, K., Thomas, L., Ferreira, M.A., Bender, D., Maller, J., Sklar, P., De Bakker, P.I., Daly, M.J. and Sham, P.C., 2007. PLINK: a tool set for whole-genome association and population-based linkage analyses. The American journal of human genetics, 81(3), pp.559-575.

## Slide 4
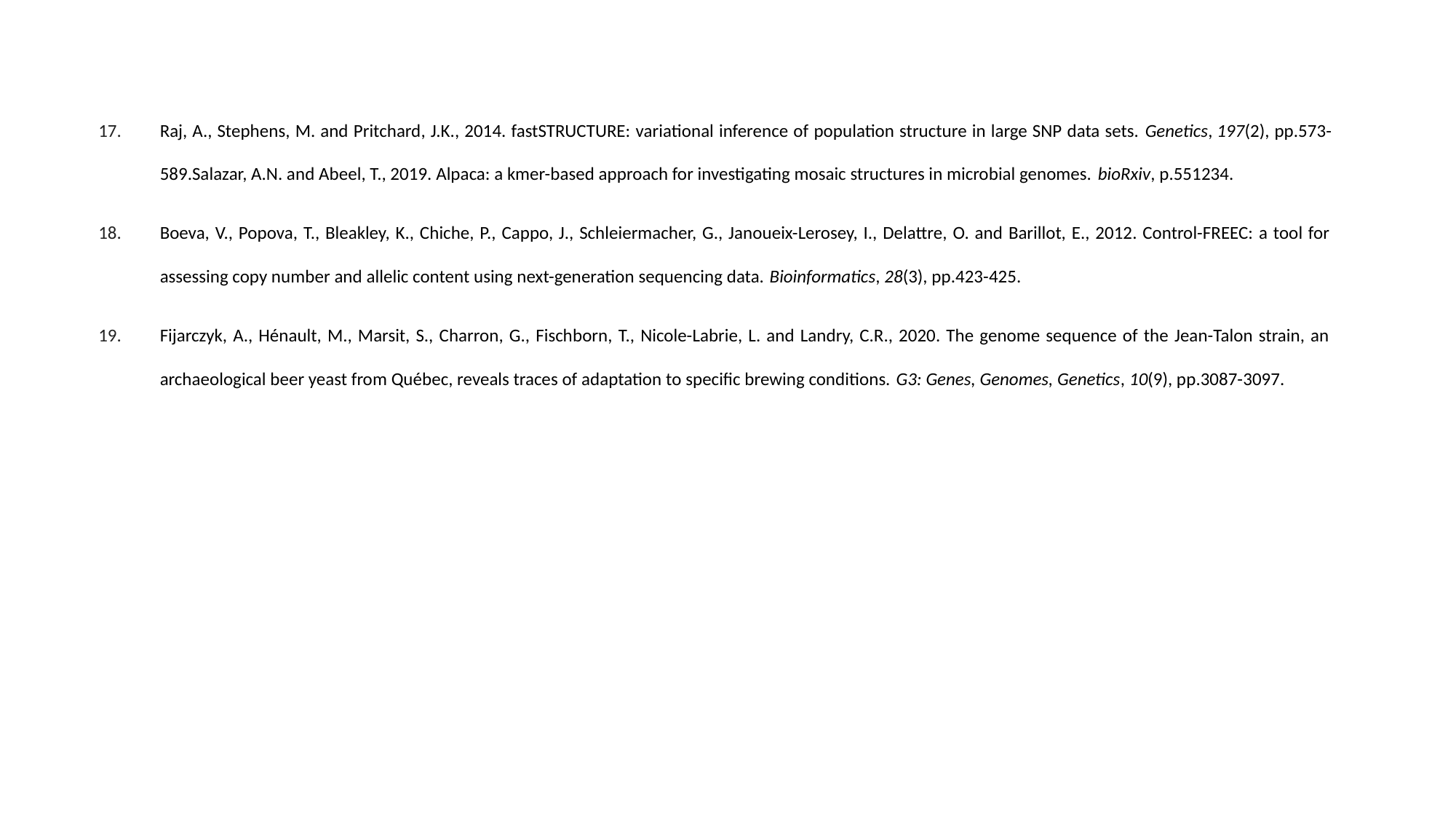

Raj, A., Stephens, M. and Pritchard, J.K., 2014. fastSTRUCTURE: variational inference of population structure in large SNP data sets. Genetics, 197(2), pp.573-589.Salazar, A.N. and Abeel, T., 2019. Alpaca: a kmer-based approach for investigating mosaic structures in microbial genomes. bioRxiv, p.551234.
Boeva, V., Popova, T., Bleakley, K., Chiche, P., Cappo, J., Schleiermacher, G., Janoueix-Lerosey, I., Delattre, O. and Barillot, E., 2012. Control-FREEC: a tool for assessing copy number and allelic content using next-generation sequencing data. Bioinformatics, 28(3), pp.423-425.
Fijarczyk, A., Hénault, M., Marsit, S., Charron, G., Fischborn, T., Nicole-Labrie, L. and Landry, C.R., 2020. The genome sequence of the Jean-Talon strain, an archaeological beer yeast from Québec, reveals traces of adaptation to specific brewing conditions. G3: Genes, Genomes, Genetics, 10(9), pp.3087-3097.
